# Reliable detection of *Burkholderia pseudomallei* using multiple cross displacement amplification label-based biosensor

**DOI:** 10.1186/s12866-022-02485-2

**Published:** 2022-03-10

**Authors:** Xiaoxia Wang, Licheng Wang, Huaxiong Zhu, Chongzhen Wang, Xiong Zhu

**Affiliations:** Central & Clinical Laboratory of Sanya People’s Hospital, Hainan 572000 Sanya, P. R. China

**Keywords:** *Burkholderia pseudomallei*, Melioidosis, Biosensor, Isothermal amplification technique, Rapid diagnosis

## Abstract

**Background:**

*Burkholderia pseudomallei* (*B. pseudomallei*), as a highly pathogenic organism, causes melioidosis, which is a disease of public health importance in many tropical developing countries. Here, we present and validate a novel detection technique, termed multiple cross displacement amplification combined with nanoparticles-based lateral flow biosensor (MCDA-NB), for identifying *B. pseudomallei* and diagnosing melioidosis.

**Results:**

*B. pseudomallei*-MCDA targets the *TTS1* (Type III secretion system gene cluster 1) to specifically design ten MCDA primers. The nanoparticles-based biosensor (NB) can be combined with *B. pseudomallei*-MCDA for visually, objective, simply and rapidly reporting reaction results. The optimal amplification conditions of *B. pseudomallei*-MCDA were 66 °C for 30 min. Assay’s sensitivity was 100 fg of genomic DNA in the pure cultures, and the analytical specificity was 100% by the examination of 257 strains, including 228 *B. pseudomallei* and 29 non-*B. pseudomallei*. As a result, the whole detection procedure was completed within 50 min, including 15 min for genomic DNA preparation, 30 min for l MCDA reaction, and 2 min for the interpretation of the results visually by biosensor.

**Conclusions:**

*B. pseudomallei*-MCDA assay is a rapid, sensitive and specific method for the detection of *B. pseudomallei*, and can be used as a potential tool for melioidosis diagnose in basic, field and clinical laboratories.

**Supplementary Information:**

The online version contains supplementary material available at 10.1186/s12866-022-02485-2.

## Background

*Burkholderia pseudomallei* (*B. pseudomallei*) is a highly pathogenic organism that is responsible for melioidosis [1]. Melioidosis is regarded as a potential emerging infectious disease, which causes significant public health burden in many tropical regions of the world [2]. Skin inoculation is considered as the most important route of infection, and ingestion of *B. pseudomallei* contaminated water and inhalation of target pathogen during extreme weather events are also important routes of infection [3]. The clinical presentation of melioidosis present highly variable in terms of disease process, severity and extent of organ involvement, which range from asymptomatic to localized skin infection, or severe pneumonia, or fulminant sepsis [4]. In severe cases, the disease progresses quickly to septic shock, acute septicemia, even to death. Thus, to control and prevention of melioidosis depends on early effective and accurate diagnosis and timely treatment.

Currently, culture-based assays remain the gold standard for the identification of *B. pseudomallei* and diagnosis of melioidosis clinically [5]. Whereas, definitive diagnosis requires a series of traditional microbiologic examinations for up to a week to complete. Although some serologic techniques, including indirect hemagglutination assay, dot immunoassay and enzyme-linked immunosorbent assay, are available for early diagnosis, they are not particularly useful in areas endemic for melioidosis because past exposure to this pathogen may yield seroconversion [6]. Moreover, *B. pseudomallei* is resistant to some antibiotics, which are used for the empirical treatment of sepsis. Herein, a rapid and reliable detection method would ensure early appropriate antibiotics therapy, and thus reduces the mortality due to delayed treatment.

Polymerase chain reaction (PCR)-based techniques, such as conventional PCR, multiplex PCR, real-time PCR and multiplex real-time PCR, have been designed for detection of *B. pseudomallei* nucleic acids, targeting several genes including the *fliC* gene, encoding flagellin, *rpsU* gene, encoding ribosomal protein subunit S21, *TTS1* and *TTS2* genes, encoding type III secretion systems, 16S rRNA, and 2 sequences (designated 8653 and 9438) unique to target bacterium [5]. Although these approaches have been validated as being useful for the detection of *B. pseudomallei*, they rely on skilled personnel, costly specialized instruments and reagents [7]. As such, these methodologies are restricted in general clinical practice [7]. To ensure management of patients and optimal therapy, advanced assays should be established for reliable, rapid and cost-effective diagnosis of this bacterium.

Isothermal amplification methodologies, as the next-generation diagnostic tools, have been widely applied for microbiological diagnosis due to their simplicity, low cost and ruggedness [8]. Up to now, only an isothermal amplification-based assay, which employed loop-mediated isothermal amplification (LAMP) as the amplification tool, was reported for the detection of *B. pseudomallei* and the diagnosis of melioidosis [9]. However, the *B. pseudomallei*-LAMP assay was limited due to the need for expensive reagents (such as calcein), expensive optical apparatus (such as real-time turbidimeter or fluorescence instrument), and agarose gel electrophoresis to analyze the method’s results [9]. Most importantly, the *B. pseudomallei*-LAMP assay exhibited poor sensitivity when compared with traditional culture-based techniques in clinical settings [9]. Herein, a rapid, simple and reliable diagnostic tool is needed for *B. pseudomallei* detection and melioidosis diagnosis.

In the present study, we employed a novel isothermal amplification assay for detecting BPSS1406 gene, which is within *TTS1* (Type III secretion system gene cluster 1) of *B. pseudomallei* and not present in the closely related species, named multiple cross displacement amplification (MCDA) [10] to rapidly detect the target bacterium and diagnose melioidosis. As a novel isothermal detection technique, the MCDA method was able to rapidly amplify target nucleic acids at a constant temperature, and displayed high sensitivity, specificity and efficiency. More recently, the normal MCDA technique was combined with nanoparticles-based biosensor (NB; MCDA-NB), which further simplified the diagnostic tools [7, 11]. Briefly, the approach required to design a set of 10 specially primers to span 10 distinct regions of target sequence, and was conducted under constant temperature (60–69 °C) within 40 min. The double-labeled detectable amplicons were generated from C1 and D2 primers labeled with FITC and biotin at the 5’ end, respectively. The FITC labeled at end of the detectable products could be bound by the anti-FITC body coated on the test line of the biosensor. Meanwhile, colored bands of test line were visualized by dye streptavidin-coated polymer nanoparticles bound with biotin-labeled at the other end of amplicons. Then, the excess of dye streptavidin-coated polymer nanoparticles was captured by biotinylated bovine serum albumin fixed on the control line of the biosensor for color development of control line to assure the validity of the biosensor. When using visual detection reagent, it was degraded into groups of A and B as the amplification reaction progressed, leading to the color change from light green to colorless. Once positive amplification was occurred, the amplified products could bind with group of A in detection reagent thus causing a color change in the reaction solution from colorless to light green, while the reaction solution of the negative controls and blank control without amplification kept colorless. In this study, the assay was optimized, including reaction temperature and isothermal amplification time. Then, the assay’s sensitivity, specificity and feasibility also were evaluated using pure cultures and clinical samples.

## Results

### Confirmation of *B. pseudomallei*-MCDA products

**Fig. 1:**
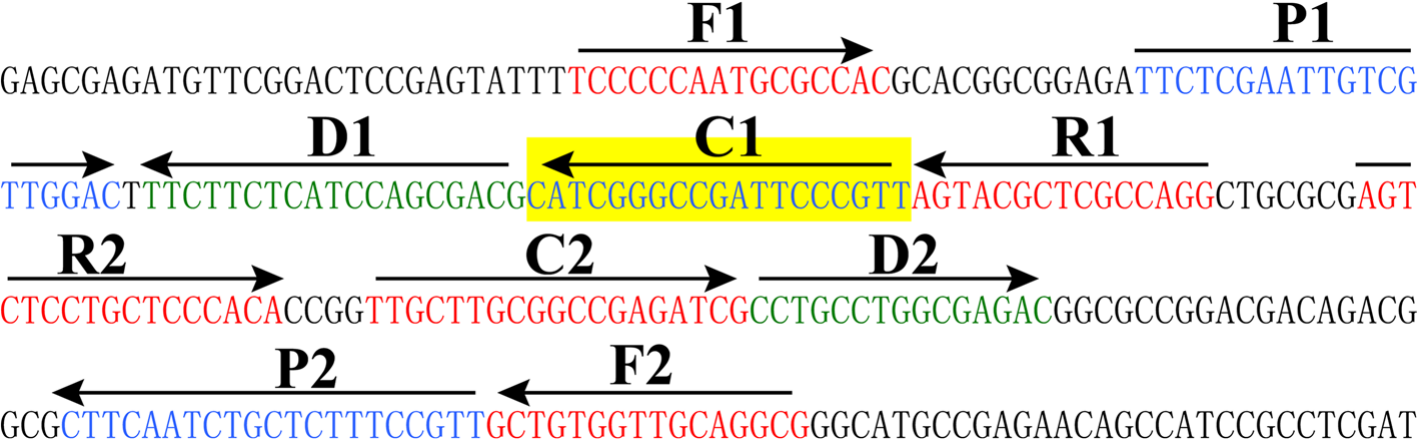
Sequence and location of target sequence BPSS1406 arranged in *TSS1* (Type III secretion system 1) used to design multiple cross displacement amplification primers. Right arrows and left arrows indicate sense and complementary sequences that are used. The positions of the primers are shown in a different color

**Fig. 2:**
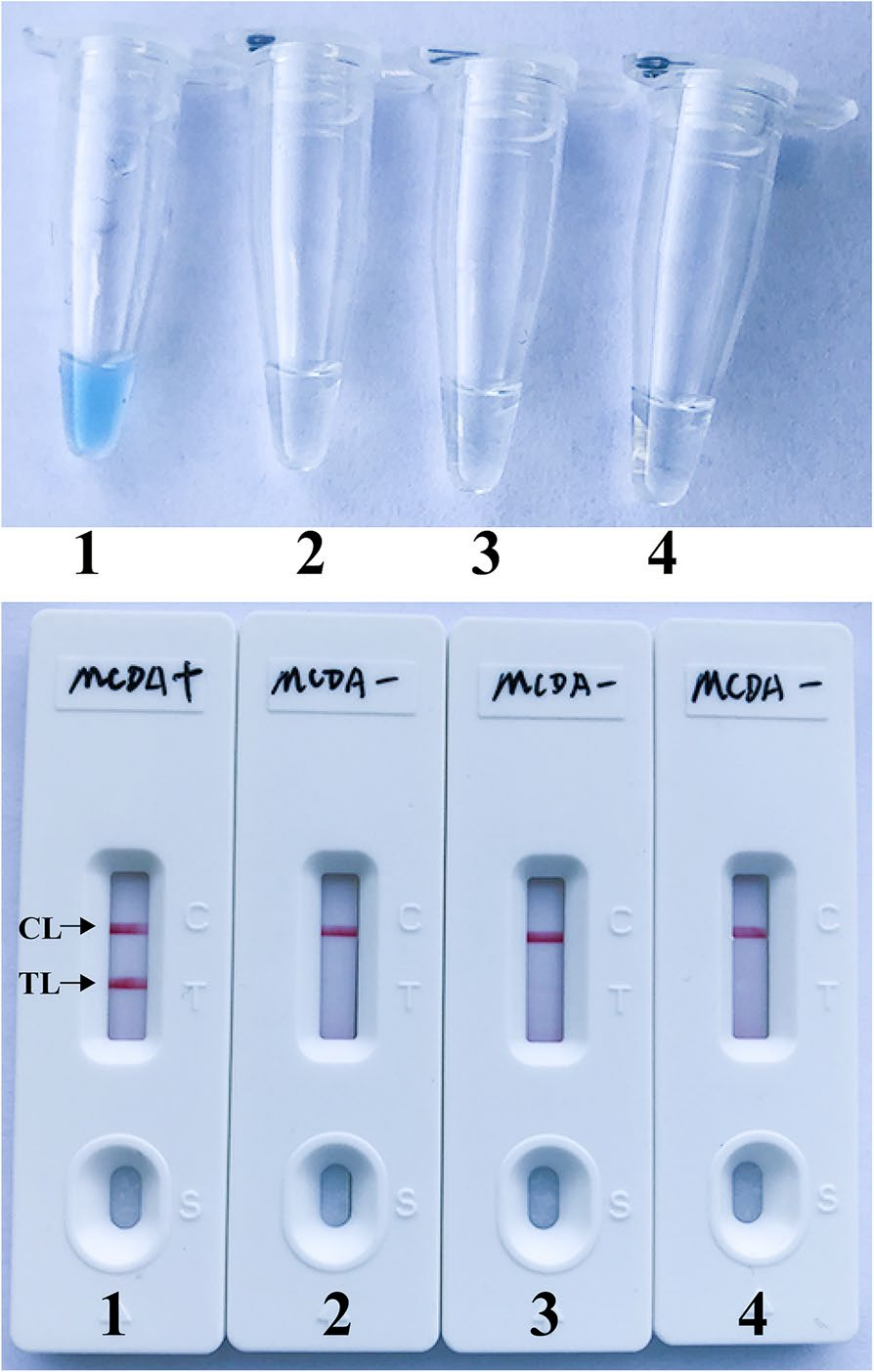
Confirmation and verification of *B. pseudomallei*-MCDA products. Color change of *B. pseudomallei*-MCDA tubes (Upper); NB applied for visual detection of *B. pseudomallei* -MCDA products (Bottom). Tube 1 (biosensor 1), positive amplification; tube 2 (biosensor 2), negative amplification (*Vibrio parahemolyticus*), tube 3 (biosensor 3), negative amplification (*Listeria monocytogenes*), tube 4 (biosensor 4), negative control (DW). TL, test line; CL, control line

The location of each MCDA primer in the target sequence was displayed in Fig. 1, and the primer sequences were shown in Table 1. The color change of reaction tube from blue to light green was observed under natural light when the *B. pseudomallei*-MCDA amplifications were positive (Fig. 2, Upper). In contrast, the color change of reaction tube from blue to colorlessness was seen under natural light when the *B. pseudomallei*-MCDA amplifications were negative (Fig. 2, Upper).

**Table 1:** The primers used in the current report

**Primers **^**a**^	**Sequences and modifications **^**b**^	**Length **^**c**^	**Genes**
F1	5’-TCCCCCAATGCGCCAC-3’	16 nt	*BPSS1406*
F2	5’-CGCCTGCAACCACAGC-3’	16 nt	
CP1	5’-AACGGGAATCGGCCCGATGTTCTCGAATTGTCGTTGGAC-3’	39 mer	
CP2	5’-TTGCTTGCGGCCGAGATCGACGGAAAGAGCAGATTGAAG-3’	39 mer	
C1	5’-AACGGGAATCGGCCCGATG-3’	19 nt	
C1*	5’-FITC-AACGGGAATCGGCCCGATG-3’	19 nt	
C2	5’-TTGCTTGCGGCCGAGATCG-3’	19 nt	
D1	5’-CGTCGCTGGATGAGAA-3’	16 nt	
D2	5’-CCTGCCTGGCGAGAC-3’	15 nt	
D2*	5’-Biotin-CCTGCCTGGCGAGAC-3’	15 nt	
R1	5’-CCTGGCGAGCGTACT-3’	15 nt	
R2	5’-AGTCTCCTGCTCCCACA-3’	17 nt	

^a^C1*, 5’-labeled with FITC when used in *B. pseudomallei*-MCDA-NB assay; D2*, 5’-labeled with Biotin when used in *B. pseudomallei*-MCDA-NB assay

^b^*FITC* fluorescein isothiocyanate

^c^*mer* monomeric unit, *nt* nucleotide

Using nanoparticles-based biosensor, the control line (CL) and test line (TL) were observed simultaneously when the positive amplification results were added into the sample pad (Fig. 2, Bottom). In contrast, only the CL was observed in the negative and blank controls (Fig. 2, Bottom). These data suggested that the MCDA primer set designed in the current study was available for establishment of *B. pseudomallei*-MCDA method for simple, rapid and reliable diagnosis of the target pathogen.

### Optimal amplification temperature for *B. pseudomallei*-MCDA assay

**Fig. 3:**
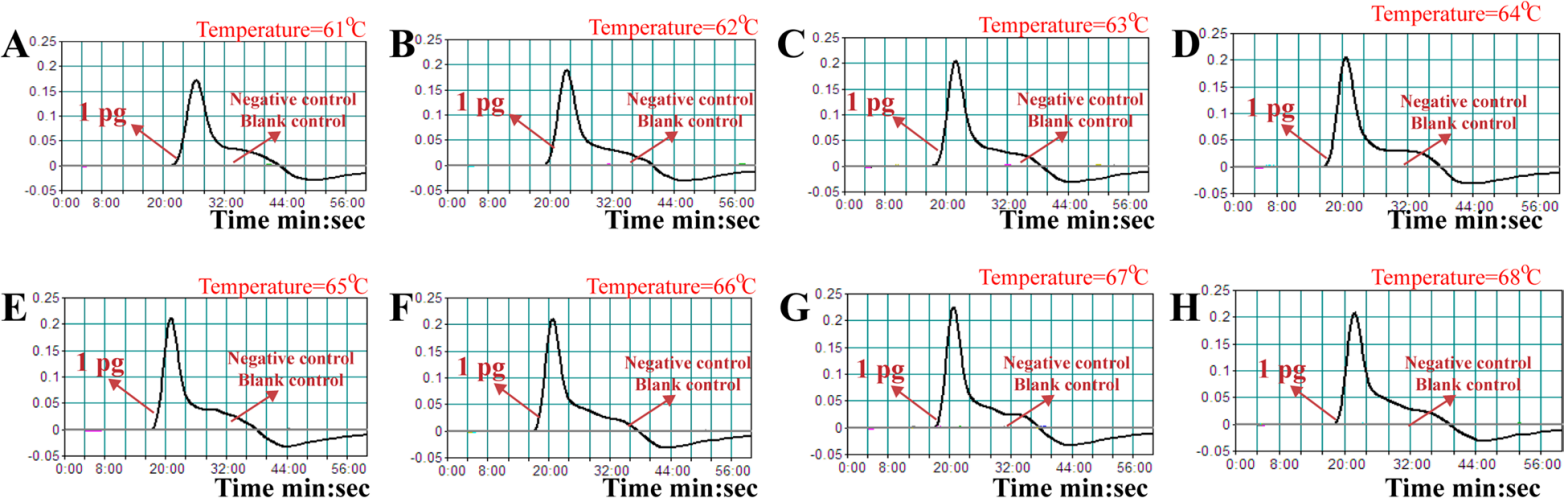
Optimal temperature for *B. pseudomallei-*MCDA primer set. Eight kinetic graphs (1-8) were obtained at various temperatures (61°C-68°C, 1°C intervals) with target pathogens DNA at the level of 1 pg per tube. The graphs from (64°C) to (67°C) showed robust amplification. The threshold value was 0.1 and the turbidity of >0.1 was considered to be positive

Different temperatures ranging from 61ºC to 68ºC with 1ºC intervals were performed and compared under the standard protocol presented above, and the monitoring technique (real-time turbidity analysis) was applied for indicating the *B. pseudomallei*-MCDA results. As shown in Fig. 3, all reaction temperatures generated the kinetics graphs, and the faster amplification reactions were seen from the assay temperature of 64ºC to 67ºC. In this report, the amplification temperature of 66ºC was the optimal temperature condition for *B. pseudomallei*-MCDA assay, and was employed for the rest of the experiments.

### Sensitivity of *B. pseudomallei*-MCDA assay

**Fig. 4:**
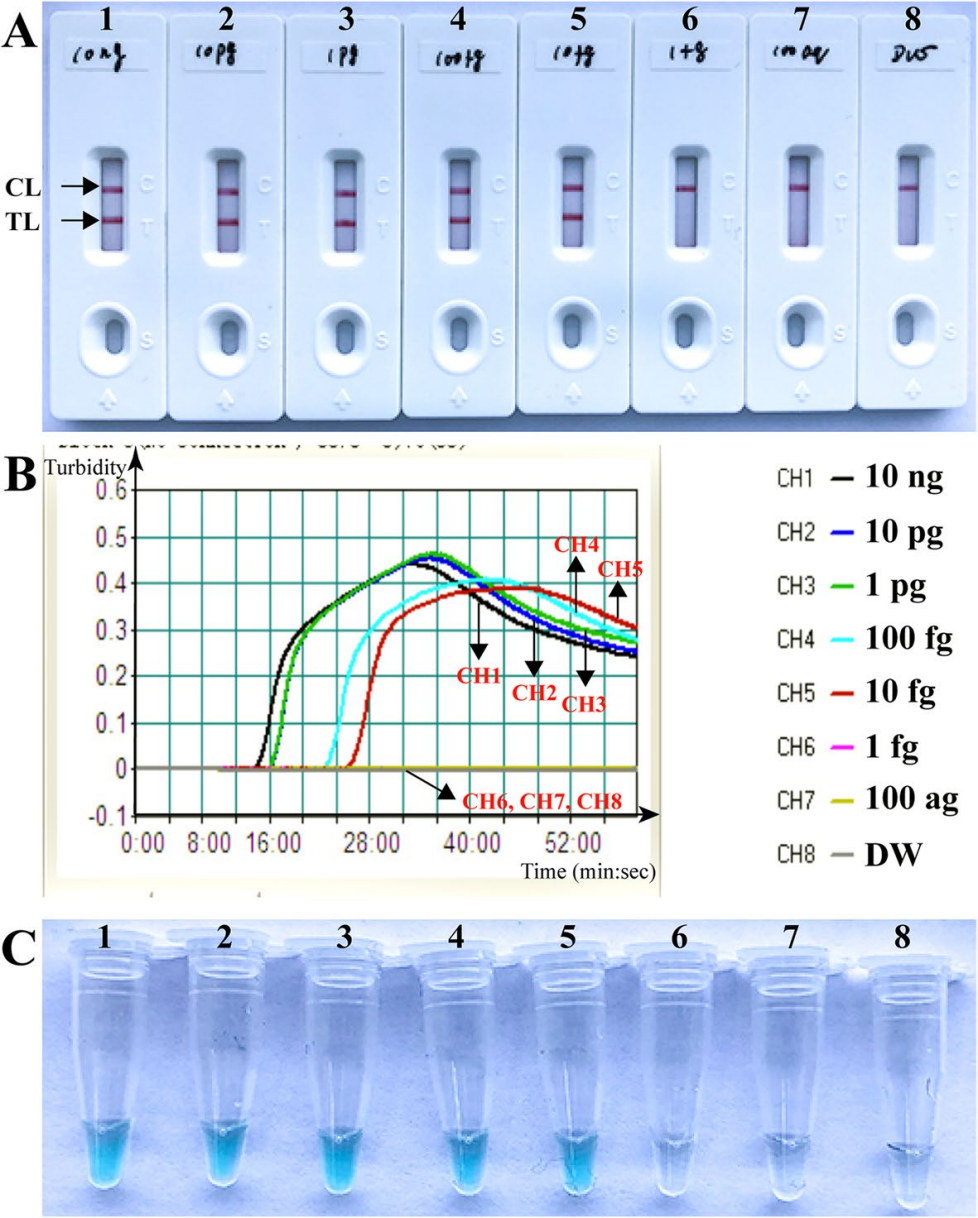
Analytical sensitivity of MCDA-NB assay using serially diluted genomic templates with *B. pseudomallei*-MCDA-NB strain (BPC006). Biosensors (**A**)/Signals (**B**)/Tubes (**C**) 1-8 represented the DNA levels of 10 ng, 10 pg, 1 pg, 100 fg, 10 fg, 1 fg, 100 ag per reaction and blank control (DW). The genomic DNA levels of 10 ng to 10 fg per reaction produced the positive reactions

The *B. pseudomallei*-MCDA developed in this report can detect down to 10 fg of target templates per reaction (Fig. 4). Using biosensor, TL and CL simultaneously presented at the detection region (Fig. 4A). In particular, the limit of detection (LoD) observed in biosensor was in good agreement with VDR and real-time turbidity analysis (Fig. 4B and C).

### Optimal amplification time for *B. pseudomallei*-MCDA assay

**Fig. 5:**
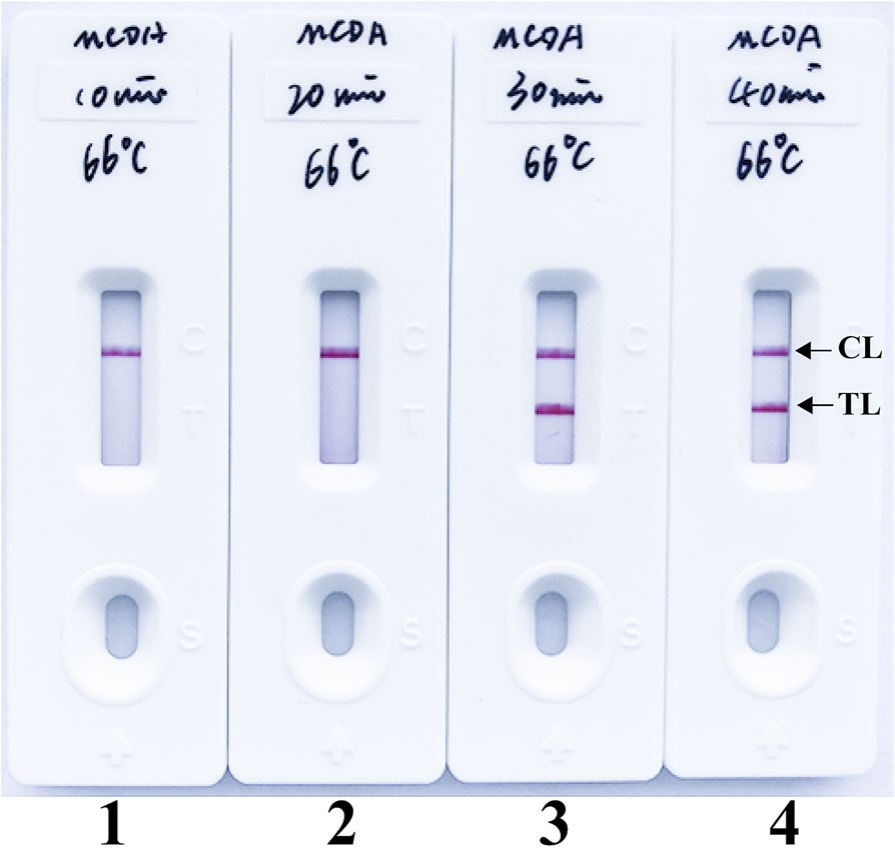
Optimal duration of time required for *B. pseudomallei*-MCDA-NB assay. Four different reaction times (Biosensor 1, 10 min; Biosensor 2, 20 min; Biosensor 3, 30 min; and Biosensor 4, 40 min) were examined and compared at 66°C. *B. pseudomallei*-MCDA reactions were performed using the LoD level of templates (10 fg per reaction), and the templates at the LoD level were able to been detected when the isothermal amplification only lasted for 30 min (Biosensor 3)

The optimal amplification time of *B. pseudomallei*-MCDA method was determined by testing at 4 different amplification times (10 min to 40 min, with 10 intervals). As shown in Fig. 5, the lowest level of genomic DNA (10 fg) can produce positive results when the *B. pseudomallei*-MCDA reaction only lasted 30 min at 66ºC. The assay time of 30 min was used as the optimal reaction time and used for the following experiments. Thus, the whole detection process, which consists of rapid DNA extraction (15 min), isothermal amplification (30 min) and result indicating (2 min), can be finished within 50 min.

### Specificity of *B. pseudomallei*-MCDA assay

**Fig. 6:**
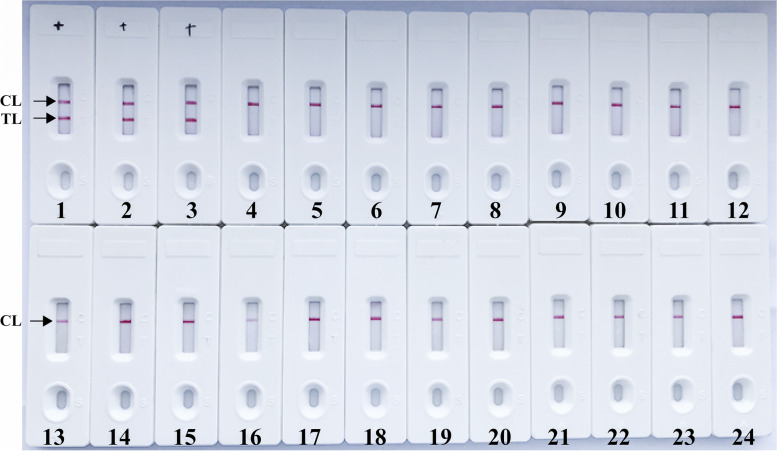
Analytical specificity of *B. pseudomallei*-MCDA-NB assay using different bacterial strains. The *B. pseudomallei*-MCDA-NB was evaluated using different genomic DNA templates. Biosensor 1, *B. pseudomallei* (BPC006); biosensor 2, *B. pseudomallei* (BP053); biosensor 3, *B. pseudomallei* (BP069); biosensor 4, *B. thailandensis*; biosensor 5, *B. cepacia*; biosensor 6-23, *Pseudomonas aeruginosa*; *Klebsiella pneumoniae*; *Staphylococcus aureus*; *Bacillus cereus*; *Staphylococcus epidermidis*; *Candida tropicalis*; *Candida albicans*; *Staphylococcus saprophytics*; *Enterotoxigenic E. coli*; *Salmonella*; *Shigella flexneria*; *Listeria monocytogenes*; *Streptococcus pneumonia*; *Staphylococcus suis*; *Enterococcus faecalis*; *Enterococcus faecium*; *Vibrio parahemolyticus*; *Stenotrophomonas maltophilia*; biosensor 24, negative control (DW)

The analytical specificity of the target technique was determined using genomic DNAs extracted from various strains (Table 2). After a 30-min isothermal reaction at 66ºC, only the genomic DNAs extracted from *B. pesudomallei* yielded positive results (Table 2 and Fig. 6), and both the test and control line positions turn red in the detection regions of biosensor. Only the control line appeared in analysis areas of biosensor, which reported the negative amplification for blank control and non-*B. pseudomallei* strains (Fig. 6). These results suggested that *B. pesudomallei*-MCDA method can correctly detect *B. pseudomallei*, and no cross-reactions were obtained from non-*B. pesudomallei* templates.

**Table 2:** Bacterial strains used in this report

**Bacteria**	**Source of strains (Strain no.) **^**a**^	**Region of isolation**	**No. of strains**	**MCDA-NB result **^**b**^
*Burkholderia species*				
*B. pseudomallei*	Isolated strain from human (BPC006)	Sanya of Hainan province, China	1	P
*B. pseudomallei*	Isolated strains from human	(See Table S1)	227	P
*B. thailandensis*	Isolated strains from environment	Sanya of Hainan province, China	8	N
*B. cepacia*	Isolated strains from human	Sanya of Hainan province, China	3	N
*Non-Burkholderia species*		Sanya of Hainan province, China		
*Pseudomonas aeruginosa*	Isolated strain from human	Sanya of Hainan province, China	1	N
*Klebsiella pneumoniae*	Isolated strain from human	Sanya of Hainan province, China	1	N
*Staphylococcus aureus*	Isolated strain from human	Sanya of Hainan province, China	1	N
*Bacillus cereus*	Isolated strain from human	Sanya of Hainan province, China	1	N
*Staphylococcus epidermidis*	Isolated strain from human	Sanya of Hainan province, China	1	N
*Candida tropicalis*	Isolated strain from human	Sanya of Hainan province, China	1	N
*Candida albicans*	Isolated strain from human	Sanya of Hainan province, China	1	N
*Staphylococcus saprophytics*	Isolated strain from human	Sanya of Hainan province, China	1	N
*Enterotoxigenic E. coli*	Isolated strain from human	Sanya of Hainan province, China	1	N
*Salmonella*	Isolated strain from human	Sanya of Hainan province, China	1	N
*Shigella flexneria*	Isolated strain from human	Sanya of Hainan province, China	1	N
*Listeria monocytogenes*	ATCC EGD-e	/	1	N
*Streptococcus pneumonia*	ATCC 700674	/	1	N
*Staphylococcus suis*	Isolated strain from human	Sanya of Hainan province, China	1	N
*Enterococcus faecalis*	ATCC 35667	/	1	N
*Enterococcus faecium*	Isolated strain from human	Sanya of Hainan province, China	1	N
*Vibrio parahemolyticus*	Isolated strain from human	Sanya of Hainan province, China	1	N
*Stenotrophomonas maltophilia*	Isolated strains from human	Sanya of Hainan province, China	1	N

^a^*ATCC* American Type Culture Collection

^b^*P* positive, *N*, negative. Only *B. pseudomallei* strains could be detected by the *B. pseudomallei*-MCDA-NB technique, indicating the extremely high specificity of the assay

### Evaluation of the *B. pseudomallei*-MCDA assay using clinical samples

In order to further evaluate the feasibility of *B. pseudomallei*-MCDA, a total of 38 clinical samples of human suspected from melioidosis were examined using *B. pseudomallei*-MCDA assay and conventional culture-biotechnical technique. The data was shown in Table 3. In the case of whole clinical samples, 3 (7.89%) and 3 (7.89%) clinical samples were *B. pseudomallei*-positive by *B. pseudomallei-*MCDA and culture-based assay. The diagnosis accuracy of *B. pseudomallei*-MCDA was 100% when compared to the traditional culture-based assay.

**Table 3:** Comparison of culture-biotechnical and *B. pseudomallei*-MCDA-NB assays for the detection of *B. pseudomallei* in clinical samples

Detection methods	Clinical samples (*n*=38)	
	Positive	Negative
Culture	3	35
MCDA-NB	3	35

## Discussion

*Burkholderia pseudomallei* is a highly pathogenic organism, which is responsible for melioidosis [3]. Due to the inadequacy of traditional bacterial detection techniques and diverse clinical manifestations, melioidosis can be very difficult to diagnose [12]. Moreover, *B. pseudomallei* is intrinsically resistant to a large number of antimicrobials, and ineffective treatment may result in case fatality rates exceeding 70% [13]. Hence, methods for rapid, sensitive and reliable detection of *B. pseudomallei* are extremely needed for timely recognition of melioidosis in patients, which is able to help healthcare workers to select the appropriate antibiotics to treat the infected patients and control the disease.

To achieve such a diagnostic tool, a MCDA-NB (multiple cross displacement amplification combined with nanoparticles-based biosensor) assay targeting the type III secretion system gene cluster 1 (*TTS1*) was successfully developed for rapidly, simply and accurately detecting *B. pseudomallei* strains. According to the technical principle (Fig. 1), the MCDA primer set that specifically spans ten regions of the target sequence BPSS1406 located on the *TTS1* was designed to ensure a high specific detection of target pathogens. To demonstrate the assay’s specificity, nucleic acid templates extracted from *B. pseudomallei* and non-*B. pseudomallei* strains (Table 2) were successfully tested (Fig. 6). Our data showed that MCDA-NB assay targeting the *TTS1* detected the *B. pseudomallei* strains with 100% specificity, because amplification products were positive only from all *B. pseudomallei* templates but not from non-*B.pseudomallei* DNAs.

The nanoparticles-based biosensor (NB) was employed for indicating the reaction results, due to its simplicity of use, rapid results, easy-to- operate in routine clinical practice. To compare with the traditional monitoring technique (i.e., real-time turbidity and colorimetric indicator) used in this report (Figs. 2 and 3), analysis of reaction products by NB was faster, simpler and less error-prone. Moreover, indicating of *B. pseudomallei*-MCDA results using NB did not rely on special reagent, apparatus and addition processes, thus NB was more suitable for visual, simple and rapid report of *B. pseudomallei*-MCDA results.

In this study, the sensitivity of the MCDA-NB assay devised here for identifying *B. pseudomallei* was 10 fg of DNAs in pure cultures, and the LoD observed in NB was consistent with VDR and real-time turbidity analysis (Fig. 4). In order to further test the applicability of MCDA-NB diagnosis to target pathogen, a total of 38 clinical samples were diagnosed using both *B. pseudomallei*-MCDA and culture-based technique. The diagnosis accuracy of *B. pseudomallei*-MCDA was 100% when compared to the traditional culture-based assay. However, the definitive culture-based identification relied on a battery of microbiological examinations that need up to a week to complete. In contrast, *B. pseudomallei*-MCDA assay could be conducted with extremely simple instruments (i.e., a conventional laboratory bath or heat block) that provided a constant temperature of 66ºC, avoiding the use of complex apparatus and eliminating the long turnaround times.

In conclusion, a MCDA-NB technique for detection of *B. pseudomallei* strains based on *TTS1* was successfully developed and verified using pure cultures and clinical samples. The targeting technique showed extremely high selectivity for *B. pseudomallei* detection, and the assay’s sensitivity was found to be 10 fg of DNA per vessel with pure culture. Using NB (Nanoparticle-based biosensor), the reaction results could be visually, objectively and rapidly indicated, which did not require any special reagents, instruments, or additional procedure, and was simple, disposable and easy to use. Herein, MCDA-NB assay devised here was a simple, reliable and sensitive method to rapidly identify *B. pseudomallei*, and could be employed as a valuable diagnostic tool for melioidosis in basic, field and clinical laboratory.

## Conclusions

*B. pseudomallei*-MCDA-NB for detection of *B. pseudomallei* strains was successfully developed. It showed extremely high specificity and the assay’s sensitivity was found to be 10 fg of DNA per vessel with pure culture. The whole detection procedure was completed within 50 min, which was simple, disposable and easy to use. Herein, *B. pseudomallei*-MCDA-NB devised here could be employed as a valuable diagnostic tool for melioidosis in basic, field and clinical laboratory.

## Methods

### Reagents and apparatus

Genomic DNA of each isolate was extracted with the DNA extraction kits (QIAamp DNA mini kits; Qiagen, Hilden, Germany). Color indicator (Visual detection reagent, VDR) and DNA isothermal amplification kit were purchased from Tian-jing Huidexing. Co., Ltd. (Beijing, China). The materials, including backing card, sample pad, conjugate pad, nitrocellulose membrane (NC) and absorbent pad, were obtained from the Jie-Yi Biotechnology. Co., Ltd. (Shanghai, China). Biotin-BSA (Biotinylated bovine serum albumin) and anti-FITC (rabbit anti-fluorescein antibody) were obtained from Abcam. Co., Ltd. (Shanghai, China). Dye (Crimson red) streptavidin-coated polymer nanoparticles (129 nm, 10 mg mL^−1^, 100 mM borate, pH 8.5 with 0.1% BSA, 0.05% Tween 20 and 10 mM EDTA) were purchased from Bangs Laboratories, INC. (Indiana, USA).

### Primer design

MCDA primer set targeted toward BPSS1406 gene, encoding a hypothetical protein, located on the *TTS1* gene cluster 1 (Genbank Accession Number AF074878), was designed using PP 5.0 (PPIMER PREMIER 5.0). Importantly, the specific region was not present in the closely related species, such as *B. thailandensis*, *B. cepacia* and *B. mallei* [14]. The primers pairs were synthesized by TsingKe Biotech.

### Bacterial strains and genomic DNA preparation

Firstly, *B. pseudomallei* strain BPC006 was used for establishing *B. pseudomallei*-MCDA [15], then further 239 strains including 3 *Burkholderia* species were used for validation in the laboratory (Table 2). Moreover, 18 non-*Burkholderia* strains also were applied for testing the *B. pseudomallei*-MCDA assay. The information of all isolates including strains names, source and region of isolation are summarized in Table 2. DNA was extracted from these strains by genomic DNA extraction kit. In particular, the strain *B. pseudomallei* BPC006 was employed for optimizing the conditions, including amplification confirmation, optimal amplification temperature, optimal reaction time and analytical sensitivity.

### Preparation and operation nano-particles biosensor

Nano-particles-based biosensor (NB), which was used for the MCDA results interpretation, was constructed according to previous studies [11, 16]. Briefly, NB includes 5 components named as a backing pad, an absorbent pad, an immersion pad, a conjugate pad and NC membrane. SA-NPs (Dye streptavidin-coated polymer nanoparticles) in 0.01 M PBS (PH7.4) were immobilized at the conjugate pad. The Biotin-BSA (4 mg/ml) and anti-FITC (0.15 mg/ml) in 0.01 M phosphate-buffered saline (PBS, PH7.4) were use as capture reagents and respectively immobilized at the test line (TL) and control line (CL) of NC membrane, with each line separated by 5 mm. The assembled biosensors were stored dry at the room temperature until use.

### *B. pseudomallei*-MCDA reactions

*B. pseudomallei*-MCDA reactions were performed in a 25 μl reaction system include 12.5 μl 2 × of the supplied buffer (Isothermal amplification® kits), 0.4 μM each of F1 and F2, 0.8 μM each of C1*, C2, R1, R2, D1 and D2*, 1.6 μM each of CP1 and CP2, 1 μl (8 U) of *Bst* 2.0 polymerase and DNA template (1 μl for culture strains, and 5 μl for clinical samples). VDR, real-time turbidity (LA-320C) and NB analysis were used for confirming, demonstrating and indicating the *B. pseudomallei*-MCDA reactions.

Then, the optimal temperature of *B. pseudomallei*-MCDA primers during the reaction stage was tested using different temperatures, ranging from 61ºC to 68ºC (with 1ºC intervals). In addition to the negative control (NC), with 1 μl DNA template of *Vibrio parahemolyticus* (*V. parahemolyticus*) and *Listeria monocytogenes* (*L. monocytogenes*), a blank control (BC) reaction with 1 μl double distilled water instead of target DNA were included in each MCDA run.

### Analytical sensitivity of *B. pseudomallei*-MCDA assay

Analytical sensitivity of *B. pseudomallei*-MCDA assay was determined using the serial dilution DNA of *B. pseudomallei* (BPC006), ranging from 10 ng μl^−1^ to 100 ag μl^−1^ (10 ng, 10 pg, 1 pg, 100 fg, 10 fg, 1 fg, and 100 ag per microliter). Then, one microliter of diluted series DNA was used as a template of subsequent amplification to determine the limit of detection (LOD). The detection limit of *B. pseudomallei*-MCDA assay was defined as the final dilution with each positive reaction, which was assessed by visual detection reagent, nanoparticle-based lateral flow biosensor and a real-time turbidimeter (LA-320C).

### Optimal reaction time for *B. pseudomallei*-MCDA assay

In this report, the optimal reaction time for *B. pseudomallei*-MCDA assay was assessed for different reaction times (ranging from 10 to 40 min with 10 intervals), then were examined and compared at the optimal conditions described above. The *B. pseudomallei*-MCDA results were reporting using NB.

### Analytical specificity of *B. pseudomallei*-MCDA assay

The assay’s specificity was determined using *B. pseudomallei* and non-*B. pseudomallei* strains with DNA concentration at least 10 ng/μl (Table 2). All amplification products were checked by the nanoparticles-based biosensor.

### Examination of the feasibility of *B. pseudomallei*-MCDA assay

After culture testing, the excess clinical samples were collected in patients with suspected melioidosis, who seen at Sanya People’s Hospital, Hainan, between 18 Jan to 30 Jun 2021. Clinical samples included blood, sputum/trachea aspirate, pus and wound swabs. DNA was extracted directly from blood, sputum/trachea aspirate and pus samples with volume of 200 μl. Swabs were submerged in 500 μl of sterile distilled water for 10 min and vortexed for 3 min, and 200 μl of this was used for DNA extraction. The specimens were firstly diagnosed in the Sanya People’s Hospital, and the identity of *B. pseudomallei* isolates was confirmed by screening for morphology in Gram staining, colonial morphology on blood agar, oxidase reaction, and COMPACT VITEK2 (BioMerieux Ltd., France) identification system [9]. Which The DNA templates were directly extracted within 2 h of clinical samples collection according to the manufacturer’s instructions. A volume of 5 μl DNA was used as templates for *B. pseudomallei*-MCDA assay. The comparisons of detection results were made between MCDA assay and culture-based test.

## Supplementary Information

**Additional file 1: Table S1.** Strain information of *Burkholderia pseudomallei* used in this report.

## Data Availability

We declared that materials described in the manuscript, including all relevant raw data, will be available from the corresponding author on reasonable request. The GenBank/ EMBL/ DDBJ accession number for the type III secretion system gene cluster 1 sequence is AF074878.

## Abbreviations

MCDA: Multiple cross displacement amplification
TTS1: Type III secretion system 1
NB: Nanoparticles-based biosensor
LAMP: Loop-mediated isothermal amplification
NC: Nitrocellulose
TL: Test line
CL: Control line
Biotin-BSA: Biotinylated bovine serum albumin
anti-FITC: Rabbit anti-fluorescein antibody
ATCC: American Type Culture Collection
DW: Distilled water
VDR: Visual detection reagent
