# Supplementary material for: Reliable detection of Burkholderia pseudomallei using multiple cross displacement amplification label-based biosensor
Source: BMC Microbiol. 2022 Mar 10;22:72. doi: 10.1186/s12866-022-02485-2 (PMC8908694; doi:10.1186/s12866-022-02485-2)
Supplement: Supplementary file 1 — Additional file 1: Table S1. Strain information of Burkholderia pseudomallei used in this report. [file 12866_2022_2485_MOESM1_ESM.docx]

**Table S1. Strain information of *Burkholderia pseudomallei* used in this report**

| **Strain number** | **Clinical sources of** **isolation** | **Years of isolation** | **Region of isolation** |
| --- | --- | --- | --- |
| BPC006 | Blood | 2009 | Sanya of Hainan province, China |
| BP001 | Sputum | 2006 | Haikou of Hainan province, China |
| BP002 | Blood | 2007 | Haikou of Hainan province, China |
| BP003 | Sputum | 2008 | Haikou of Hainan province, China |
| BP004 | Blood | 2009 | Haikou of Hainan province, China |
| BP005 | Pus | 2009 | Haikou of Hainan province, China |
| BP006 | Blood | 2009 | Haikou of Hainan province, China |
| BP007 | Blood | 2009 | Haikou of Hainan province, China |
| BP008 | Blood | 2009 | Haikou of Hainan province, China |
| BP009 | Blood | 2009 | Haikou of Hainan province, China |
| BP010 | Pus | 2009 | Haikou of Hainan province, China |
| BP011 | Blood | 2009 | Haikou of Hainan province, China |
| BP012 | Blood | 2009 | Haikou of Hainan province, China |
| BP013 | Blood | 2010 | Haikou of Hainan province, China |
| BP014 | Blood | 2010 | Haikou of Hainan province, China |
| BP015 | Blood | 2010 | Haikou of Hainan province, China |
| BP016 | Pus | 2010 | Haikou of Hainan province, China |
| BP017 | Blood | 2010 | Haikou of Hainan province, China |
| BP018 | Sputum | 2010 | Haikou of Hainan province, China |
| BP019 | Blood | 2010 | Haikou of Hainan province, China |
| BP020 | Blood | 2010 | Haikou of Hainan province, China |
| BP021 | Blood | 2010 | Haikou of Hainan province, China |
| BP022 | Blood | 2011 | Haikou of Hainan province, China |
| BP023 | Blood | 2011 | Haikou of Hainan province, China |
| BP024 | Pus | 2011 | Haikou of Hainan province, China |
| BP025 | Blood | 2010 | Haikou of Hainan province, China |
| BP026 | Blood | 2011 | Haikou of Hainan province, China |
| BP027 | Blood | 2011 | Haikou of Hainan province, China |
| BP028 | Blood | 2011 | Haikou of Hainan province, China |
| BP029 | Blood | 2011 | Haikou of Hainan province, China |
| BP030 | Blood | 2011 | Haikou of Hainan province, China |
| BP031 | Sputum | 2011 | Haikou of Hainan province, China |
| BP032 | Blood | 2011 | Haikou of Hainan province, China |
| BP033 | Blood | 2011 | Haikou of Hainan province, China |
| BP034 | Blood | 2011 | Haikou of Hainan province, China |
| BP035 | Sputum | 2011 | Haikou of Hainan province, China |
| BP036 | Blood | 2011 | Haikou of Hainan province, China |
| BP037 | Blood | 2011 | Haikou of Hainan province, China |
| BP038 | Blood | 2012 | Haikou of Hainan province, China |
| BP039 | Blood | 2012 | Haikou of Hainan province, China |
| BP040 | Sputum | 2012 | Haikou of Hainan province, China |
| BP041 | Sputum | 2012 | Haikou of Hainan province, China |
| BP042 | Sputum | 2012 | Haikou of Hainan province, China |
| BP043 | Sputum | 2012 | Haikou of Hainan province, China |
| BP044 | Pus | 2012 | Haikou of Hainan province, China |
| BP045 | Blood | 2012 | Haikou of Hainan province, China |
| BP046 | Blood | 2012 | Haikou of Hainan province, China |
| BP047 | Blood | 2012 | Haikou of Hainan province, China |
| BP048 | Blood | 2012 | Haikou of Hainan province, China |
| BP049 | Blood | 2012 | Haikou of Hainan province, China |
| BP050 | Blood | 2012 | Haikou of Hainan province, China |
| BP051 | Blood | 2012 | Haikou of Hainan province, China |
| BP052 | Blood | 2012 | Haikou of Hainan province, China |
| BP053 | Blood | 2012 | Haikou of Hainan province, China |
| BP054 | Sputum | 2012 | Haikou of Hainan province, China |
| BP055 | Blood | 2012 | Haikou of Hainan province, China |
| BP056 | Blood | 2012 | Haikou of Hainan province, China |
| BP057 | Pus | 2012 | Haikou of Hainan province, China |
| BP058 | Blood | 2012 | Haikou of Hainan province, China |
| BP059 | Blood | 2012 | Haikou of Hainan province, China |
| BP060 | Blood | 2012 | Haikou of Hainan province, China |
| BP061 | Pus | 2012 | Haikou of Hainan province, China |
| BP062 | Blood | 2012 | Haikou of Hainan province, China |
| BP063 | Sputum | 2012 | Haikou of Hainan province, China |
| BP064 | Blood | 2013 | Haikou of Hainan province, China |
| BP065 | Pus | 2013 | Haikou of Hainan province, China |
| BP066 | Sputum | 2008 | Haikou of Hainan province, China |
| BP067 | Sputum | 2009 | Haikou of Hainan province, China |
| BP068 | Sputum | 2010 | Haikou of Hainan province, China |
| BP069 | Wound swabs | 2008 | Haikou of Hainan province, China |
| BP070 | Blood | 2009 | Haikou of Hainan province, China |
| BP071 | Pus | 2009 | Haikou of Hainan province, China |
| BP072 | Pus | 2010 | Haikou of Hainan province, China |
| BP073 | Sputum | 2011 | Haikou of Hainan province, China |
| BP074 | Urine | 2011 | Haikou of Hainan province, China |
| BP075 | Blood | 2011 | Haikou of Hainan province, China |
| BP076 | Blood | 2013 | Haikou of Hainan province, China |
| BP077 | Blood | 2013 | Haikou of Hainan province, China |
| BP078 | Blood | 2013 | Haikou of Hainan province, China |
| BP079 | Blood | 2010 | Haikou of Hainan province, China |
| BP080 | Sputum | 2010 | Haikou of Hainan province, China |
| BP081 | Blood | 2013 | Haikou of Hainan province, China |
| BP082 | Pus | 2013 | Haikou of Hainan province, China |
| BP083 | Pus | 2013 | Haikou of Hainan province, China |
| BP084 | Wound swabs | 2013 | Haikou of Hainan province, China |
| BP085 | Wound swabs | 2013 | Haikou of Hainan province, China |
| BP086 | Wound swabs | 2013 | Haikou of Hainan province, China |
| BP087 | Sputum | 2013 | Haikou of Hainan province, China |
| BP088 | Pus | 2011 | Sanya of Hainan province, China |
| BP089 | Blood | 2009 | Sanya of Hainan province, China |
| BP090 | Pus | 2010 | Sanya of Hainan province, China |
| BP091 | Pus | 2010 | Sanya of Hainan province, China |
| BP092 | Blood | 2010 | Sanya of Hainan province, China |
| BP093 | Blood | 2006 | Sanya of Hainan province, China |
| BP094 | Blood | 2007 | Sanya of Hainan province, China |
| BP095 | Pus | 2002 | Sanya of Hainan province, China |
| BP096 | Blood | 2008 | Sanya of Hainan province, China |
| BP097 | Pus | 2002 | Sanya of Hainan province, China |
| BP098 | Blood | 2009 | Sanya of Hainan province, China |
| BP099 | Blood | 2008 | Sanya of Hainan province, China |
| BP100 | Blood | 2003 | Sanya of Hainan province, China |
| BP101 | Blood | 2002 | Sanya of Hainan province, China |
| BP102 | Blood | 2002 | Sanya of Hainan province, China |
| BP103 | Blood | 2002 | Sanya of Hainan province, China |
| BP104 | Blood | 2004 | Sanya of Hainan province, China |
| BP105 | Blood | 2005 | Sanya of Hainan province, China |
| BP106 | Sputum | 2006 | Sanya of Hainan province, China |
| BP107 | Pus | 2008 | Sanya of Hainan province, China |
| BP108 | Sputum | 2009 | Sanya of Hainan province, China |
| BP109 | Blood | 2009 | Sanya of Hainan province, China |
| BP110 | Pus | 2009 | Sanya of Hainan province, China |
| BP111 | Pus | 2008 | Sanya of Hainan province, China |
| BP112 | Pus | 2005 | Sanya of Hainan province, China |
| BP113 | Blood | 2002 | Sanya of Hainan province, China |
| BP114 | Blood | 2008 | Sanya of Hainan province, China |
| BP115 | Blood | 2011 | Sanya of Hainan province, China |
| BP116 | Blood | 2010 | Sanya of Hainan province, China |
| BP117 | Blood | 2011 | Sanya of Hainan province, China |
| BP118 | Blood | 2013 | Sanya of Hainan province, China |
| BP119 | Blood | 2013 | Sanya of Hainan province, China |
| BP120 | Pus | 2013 | Sanya of Hainan province, China |
| BP121 | Pus | 2013 | Sanya of Hainan province, China |
| BP122 | Blood | 2012 | Sanya of Hainan province, China |
| BP123 | Blood | 2010 | Sanya of Hainan province, China |
| BP124 | Blood | 2012 | Sanya of Hainan province, China |
| BP125 | Blood | 2012 | Sanya of Hainan province, China |
| BP126 | Blood | 2012 | Sanya of Hainan province, China |
| BP127 | Blood | 2011 | Sanya of Hainan province, China |
| BP128 | Pus | 2012 | Sanya of Hainan province, China |
| BP129 | Pus | 2013 | Sanya of Hainan province, China |
| BP130 | Pus | 2013 | Sanya of Hainan province, China |
| BP131 | Blood | 2013 | Sanya of Hainan province, China |
| BP132 | Cerebrospinal fluid | 2013 | Sanya of Hainan province, China |
| BP133 | Wound swabs | 2013 | Sanya of Hainan province, China |
| BP134 | Wound swabs | 2013 | Sanya of Hainan province, China |
| BP135 | Blood | 2014 | Sanya of Hainan province, China |
| BP136 | Blood | 2014 | Sanya of Hainan province, China |
| BP137 | Blood | 2014 | Sanya of Hainan province, China |
| BP138 | Sputum | 2014 | Sanya of Hainan province, China |
| BP139 | Blood | 2014 | Sanya of Hainan province, China |
| BP140 | Blood | 2014 | Sanya of Hainan province, China |
| BP141 | Blood | 2014 | Sanya of Hainan province, China |
| BP142 | Blood | 2014 | Sanya of Hainan province, China |
| BP143 | Urine | 2014 | Sanya of Hainan province, China |
| BP144 | Sputum | 2014 | Sanya of Hainan province, China |
| BP145 | Sputum | 2014 | Sanya of Hainan province, China |
| BP146 | Blood | 2014 | Sanya of Hainan province, China |
| BP147 | Blood | 2014 | Sanya of Hainan province, China |
| BP148 | Sputum | 2014 | Sanya of Hainan province, China |
| BP149 | Blood | 2014 | Sanya of Hainan province, China |
| BP150 | Blood | 2014 | Sanya of Hainan province, China |
| BP151 | Sputum | 2015 | Sanya of Hainan province, China |
| BP152 | Sputum | 2015 | Sanya of Hainan province, China |
| BP153 | Sputum | 2015 | Sanya of Hainan province, China |
| BP154 | Blood | 2015 | Sanya of Hainan province, China |
| BP155 | Blood | 2015 | Sanya of Hainan province, China |
| BP156 | Wound swabs | 2015 | Sanya of Hainan province, China |
| BP157 | Blood | 2015 | Sanya of Hainan province, China |
| BP158 | Sputum | 2015 | Sanya of Hainan province, China |
| BP159 | Urine | 2015 | Sanya of Hainan province, China |
| BP160 | Blood | 2015 | Sanya of Hainan province, China |
| BP161 | Blood | 2015 | Sanya of Hainan province, China |
| BP162 | Blood | 2015 | Sanya of Hainan province, China |
| BP163 | Blood | 2015 | Sanya of Hainan province, China |
| BP164 | Pus | 2015 | Sanya of Hainan province, China |
| BP165 | Pus | 2016 | Sanya of Hainan province, China |
| BP166 | Blood | 2016 | Sanya of Hainan province, China |
| BP167 | Blood | 2016 | Sanya of Hainan province, China |
| BP168 | Sputum | 2016 | Sanya of Hainan province, China |
| BP169 | Blood | 2016 | Sanya of Hainan province, China |
| BP170 | Blood | 2016 | Sanya of Hainan province, China |
| BP171 | Blood | 2016 | Sanya of Hainan province, China |
| BP172 | Blood | 2016 | Sanya of Hainan province, China |
| BP173 | Blood | 2016 | Sanya of Hainan province, China |
| BP174 | Blood | 2016 | Sanya of Hainan province, China |
| BP175 | Blood | 2016 | Sanya of Hainan province, China |
| BP176 | Blood | 2017 | Sanya of Hainan province, China |
| BP177 | Blood | 2017 | Sanya of Hainan province, China |
| BP178 | Blood | 2017 | Sanya of Hainan province, China |
| BP179 | Blood | 2017 | Sanya of Hainan province, China |
| BP180 | Sputum | 2017 | Sanya of Hainan province, China |
| BP181 | Blood | 2017 | Sanya of Hainan province, China |
| BP182 | Blood | 2017 | Sanya of Hainan province, China |
| BP183 | Sputum | 2016 | Sanya of Hainan province, China |
| BP184 | Sputum | 2016 | Sanya of Hainan province, China |
| BP185 | Blood | 2016 | Sanya of Hainan province, China |
| BP186 | Blood | 2017 | Sanya of Hainan province, China |
| BP187 | Blood | 2017 | Sanya of Hainan province, China |
| BP188 | Sputum | 2017 | Sanya of Hainan province, China |
| BP189 | Blood | 2017 | Sanya of Hainan province, China |
| BP190 | Blood | 2018 | Sanya of Hainan province, China |
| BP191 | Blood | 2018 | Sanya of Hainan province, China |
| BP192 | Blood | 2018 | Sanya of Hainan province, China |
| BP193 | Blood | 2018 | Sanya of Hainan province, China |
| BP194 | Blood | 2018 | Sanya of Hainan province, China |
| BP195 | Blood | 2018 | Sanya of Hainan province, China |
| BP196 | Blood | 2018 | Sanya of Hainan province, China |
| BP197 | Blood | 2018 | Sanya of Hainan province, China |
| BP198 | Blood | 2019 | Sanya of Hainan province, China |
| BP199 | Blood | 2019 | Sanya of Hainan province, China |
| BP200 | Wound swabs | 2019 | Sanya of Hainan province, China |
| BP201 | Blood | 2019 | Sanya of Hainan province, China |
| BP202 | Blood | 2019 | Sanya of Hainan province, China |
| BP203 | Wound swabs | 2019 | Sanya of Hainan province, China |
| BP204 | Blood | 2019 | Sanya of Hainan province, China |
| BP205 | Sputum | 2019 | Sanya of Hainan province, China |
| BP206 | Wound swabs | 2019 | Sanya of Hainan province, China |
| BP207 | Blood | 2019 | Sanya of Hainan province, China |
| BP208 | Blood | 2019 | Sanya of Hainan province, China |
| BP209 | Blood | 2020 | Sanya of Hainan province, China |
| BP210 | Blood | 2020 | Sanya of Hainan province, China |
| BP211 | Blood | 2020 | Sanya of Hainan province, China |
| BP212 | Blood | 2020 | Sanya of Hainan province, China |
| BP213 | Blood | 2020 | Sanya of Hainan province, China |
| BP214 | Blood | 2020 | Sanya of Hainan province, China |
| BP215 | Blood | 2020 | Sanya of Hainan province, China |
| BP216 | Blood | 2020 | Sanya of Hainan province, China |
| BP217 | Blood | 2020 | Sanya of Hainan province, China |
| BP218 | Blood | 2020 | Sanya of Hainan province, China |
| BP219 | Bronchoalveolar lavage fluid | 2020 | Sanya of Hainan province, China |
| BP220 | Blood | 2020 | Sanya of Hainan province, China |
| BP221 | Blood | 2020 | Sanya of Hainan province, China |
| BP222 | Blood | 2020 | Sanya of Hainan province, China |
| BP223 | Blood | 2020 | Sanya of Hainan province, China |
| BP224 | Blood | 2020 | Sanya of Hainan province, China |
| BP225 | Abscess fluid | 2021 | Sanya of Hainan province, China |
| BP226 | Blood | 2021 | Sanya of Hainan province, China |
| BP227 | Blood | 2021 | Sanya of Hainan province, China |
